# Association Between Ultraprocessed Food Consumption and Cardiovascular Disease Risk: MESA (Multiethnic Study of Atherosclerosis)

**DOI:** 10.1016/j.jacadv.2025.102516

**Published:** 2026-03-17

**Authors:** Amier Haidar, Rishi Rikhi, Karol E. Watson, Alexis C. Wood, Michael D. Shapiro

**Affiliations:** a Division of Cardiology, David Geffen School of Medicine University of California, Los Angeles, USA; b Center for Prevention of Cardiovascular Disease, Section on Cardiovascular Medicine, Department of Internal Medicine, Wake Forest University School of Medicine, Winston-Salem, North Carolina, USA; c USDA/ARS Children’s Nutrition Research Center, Baylor College of Medicine-Department of Pediatrics, Houston, Texas, USA

**Keywords:** atherosclerotic cardiovascular disease (ASCVD), diet quality, racial disparities, ultraprocessed food

## Abstract

**Background:**

Ultraprocessed foods (UPFs) have been linked to adverse cardiometabolic outcomes and increased atherosclerotic cardiovascular disease (CVD) (ASCVD) risk. However, prior research has largely focused on homogenous populations, lacking racial and ethnic diversity.

**Objectives:**

The objectives are to examine the longitudinal relationship between UPF consumption and ASCVD risk and to investigate whether these associations differ by race/ethnicity, sex, or socioeconomic status.

**Methods:**

The MESA (Multiethnic Study of Atherosclerosis) is a prospective cohort study of 6,814 U.S. adults aged 45 to 84 years, without clinically apparent CVD. UPF consumption was classified according to the Nova classification system. Multivariable cox proportional hazards models were used to evaluate the association between UPF intake and incident CVD events. Incident CVD events included nonfatal myocardial infarction, resuscitated cardiac arrest, death resulting from coronary heart disease, stroke (not transient ischemic attack), and death resulting from stroke.

**Results:**

Each additional daily serving of UPF was associated with a 5.1% increased risk of ASCVD events (HR: 1.051; 95% CI: 1.011-1.093). Participants in the highest quintile of UPF consumption had a 66.8% higher risk compared to those in the lowest (HR: 1.668; 95% CI: 1.196-2.325). A significant multiplicative interaction was observed between UPF intake and Black race (*P* = 0.010), with stratified analyses demonstrating a higher ASCVD risk in Black Americans (HR: 1.061; 95% CI: 1.016-1.108), compared to non-Black Americans (HR: 1.032; 95% CI: 1.001-1.065).

**Conclusions:**

In a large, multiethnic cohort, higher UPF consumption was significantly associated with an increased risk for ASCVD events, with a more pronounced association among Black Americans.

Healthy diets, such as the Dietary Approaches to Stop Hypertension diet or the Mediterranean diet, consisting predominately of vegetables, fruits, whole grains, nuts and legumes, and low-fat dairy have been shown to improve cardiometabolic health and reduce cardiovascular disease (CVD).^1^ In contrast, lower quality diets characterized by high intakes of red and processed meats, refined grains, added sugars, sodium, and saturated fats are associated with adverse cardiometabolic outcomes and increased risk of atherosclerotic CVD (ASCVD).^1^ Despite the risks, most Americans do not consume a healthy diet^1^ and over 50% of calorie intake comes from ultraprocessed foods (UPFs).^2^^,^^3^

UPFs are industrially manufactured formulations of food often modified by chemical processes and then assembled into ready-to-consume hyperpalatable food and drink products using flavors, colors, emulsifiers, and other cosmetic additives containing few whole foods.^4^ UPFs are defined using the Nova classification system and include foods such as soft drinks, sweet or savory packaged snacks, chocolate, candies, ice cream, packaged breads and buns, margarines and other spreads, cookies, pastries, cakes, breakfast cereals, reconstituted meat products, and more.^4^ UPFs might influence cardiometabolic health through multiple mechanisms and pathways including increasing energy intake, altering the gut microbiome, interfering with gut-brain satiety signaling, and hormonal effects which then may lead to insulin resistance, dyslipidemia, obesity, hypertension, endothelial dysfunction, and oxidative stress.^5^, ^6^, ^7^, ^8^ In addition, high UPF consumption may displace healthier, nutrient-dense foods from the diet, thereby lowering overall diet quality and further contributing to cardiometabolic risk.^3^^,^^5^

Studies have shown that high consumption of UPFs is associated with an increased ASCVD risk,^9^, ^10^, ^11^, ^12^, ^13^, ^14^, ^15^, ^16^, ^17^, ^18^, ^19^, ^20^, ^21^, ^22^, ^23^ with only a few studies reporting no association.^24^, ^25^, ^26^, ^27^ Moreover, majority of these studies were conducted in predominantly White populations or in homogenous populations outside the United States. Recently, the 2025 Dietary Guidelines Advisory Committee recommended replicating findings from observational studies conducted outside of the United States using within U.S. populations, while accounting for diversity in race/ethnicity, socioeconomic status (SES), and sex.^28^ Given longstanding racial/ethnic disparities in cardiovascular risk, there are reasons to expect heterogeneity in the UPF-CVD association across groups in the Multiethnic Study of Atherosclerosis (MESA). UPF intake differs across racial/ethnic groups^2^^,^^3^^,^^29^ and structural factors such as targeted marketing and food environments disproportionately promote UPFs in Black and Hispanic communities.^30^ Therefore, the objective of this study is to examine the longitudinal relationship between baseline UPF consumption and ASCVD risk and to investigate whether these associations differ by race/ethnicity, sex, or SES, in a large, U.S. multiethnic cohort.

## Methods

### Study design and population

MESA is a population-based, prospective cohort study of 6,814 men and women from 6 U.S. communities: Baltimore City and Baltimore County, Maryland; Chicago, Illinois; Forsyth County, North Carolina; Los Angeles County, California; Northern Manhattan and the Bronx, New York; and St. Paul, Minnesota. Baseline data were collected from July 2000 and August 2002 (exam 1). Participants ranged in age from 45 and 84 years and were free of clinically apparent CVD. The study protocol was approved by the institutional review boards at all field centers. During the baseline exam, all participants provided written informed consent and the participants were invited to complete subsequent exams and questionnaires. Follow-up exams were conducted from September 2002 through February 2004 (exam 2), March 2004 through September 2005 (exam 3), September 2005 through May 2007 (exam 4), and April 2010 through February 2012 (exam 5). Further details of the study design have previously been published.^31^

### Measurement of covariates

Standardized questionnaires were used to gather information on age, sex, race and ethnicity, education and income levels, occupational information, smoking status, medical history, physical activity, and medication use for diabetes, lipid lowering, and hypertension. Height was measured with a stadiometer and body weight with a balance scale to the nearest 0.1 cm and 0.5 kg, respectively. Waist circumference was measured to the nearest 0.1 cm, at the minimum abdominal girth using a steel measuring tape with standard 4-ounce tension. Seated blood pressure was taken 3 times at 1-min intervals following a standardized protocol. The average of the last 2 measurements were used for analysis. Total and high-density lipoprotein cholesterol and triglycerides were measured at a central laboratory (University of Vermont, Burlington, Vermont).

### Dietary assessment

At the baseline exam, participants completed a 120-item food-frequency questionnaire (FFQ) assessing frequency of consumption (times per day, week, or month) and the serving size (small, medium, or large) of each FFQ item.^32^^,^^33^ The FFQ was previously validated in non-Hispanic Whites, Hispanics, and Black Americans, and evaluated against plasma lipid concentrations within the MESA cohort.^34^, ^35^, ^36^, ^37^ Servings per day were calculated by multiplying consumption frequency by the reported serving size.^34^

UPFs were categorized according to the Nova classification system^4^ and a systematic, multistep process was followed to ensure reproducibility and accuracy. First, 2 investigators independently reviewed all FFQ items and assigned them to 1 of the 4 Nova categories based on standard definitions. Second, the classifications were compared, and any discrepancies were discussed and resolved through consensus, with adjudication by a senior investigator when needed. The following foods were categorized as ultraprocessed: sausage, pancakes, hot cereal, cold cereal, white bread, dark bread, muffins, biscuits, margarine on rolls, chips, crackers, french fries, oriental noodles, dumplings, chow mein, refried beans, pizza, hamburger, hamhocks, fried chicken, fried fish, ice cream, frozen yogurt, tofu dessert, white donuts, chocolate donuts, pies, pudding, candy, sweet milk, soy milk, soda, diet soda, instant breakfast, hot cocoa, and liquor. The servings per day of each food item were totaled to determine the total amount of UPF consumed for each participant. Each item was mapped to the Nova classification system based on its typical formulation and processing characteristics, using generic examples to illustrate the industrial nature of these products (Supplemental Table 2).

Diet quality was assessed using the Alternative Healthy Eating Index (AHEI), a dietary score developed to capture foods and nutrients consistently linked to chronic disease risk.^38^, ^39^, ^40^, ^41^, ^42^, ^43^ The AHEI assigns 0 to 10 points for each component, with higher scores reflecting greater consumption of vegetables, fruits, whole grains, nuts and legumes, long-chain omega-3 and polyunsaturated fatty acids, and moderate alcohol intake, as well as lower consumption of sugar-sweetened beverages, red and processed meats, trans fats, and sodium.^38^^,^^44^^,^^45^ Total scores range from 0 (minimal adherence) to 110 (optimal adherence). For analysis, participants were classified as having a healthy diet if their AHEI score fell within the top quintile of the cohort distribution, consistent with prior studies showing that high AHEI scores are associated with a substantially lower risk of CVD.^38^^,^^44^^,^^45^

### CVD events

Events and mortality were verified with medical records or interviews by 2 physicians from MESA. Incident hard CVD events included nonfatal myocardial infarction (MI), resuscitated cardiac arrest, death resulting from CHD, stroke (not transient ischemic attack), and death resulting from stroke. Incident CVD events were adjudicated by the MESA mortality and morbidity review committee. MI required either abnormal cardiac biomarkers (more than 2 times upper limits of normal) regardless of chest pain or electrocardiogram findings; evolving Q waves regardless of chest pain or biomarker findings; or a combination of chest pain, and ST-T evolution or new left bundle branch block, and biomarker levels 1 to 2 times upper limits of normal. Resuscitated cardiac arrest was classified when a patient successfully recovered from a full cardiac arrest through cardiopulmonary resuscitation. Fatal CHD required a documented MI within the previous 28 days, chest pain within the 72 h before death, or a history of CHD, and required the absence of a known nonatherosclerotic or noncardiac cause of death. Neurologists reviewed and classified stroke as present if there was a focal neurologic deficit lasting 24 hours or until death, with a clinically relevant lesion on brain imaging, and no nonvascular cause.

### Statistical analysis

Descriptive statistics were described with continuous variables presented as mean (SD) or median (IQR) based on distribution and categorical variables presented as frequencies (percentages). Baseline characteristics were compared across quintiles of UPF consumption using chi-square tests for categorical variables and analysis of variance or Kruskal-Wallis tests for continuous variables. To assess the stability of UPF intake over time, we examined mean UPF consumption among participants with available dietary data at both exam 1 (2000-2002) and exam 5 (2010-2012). We calculated the difference in mean UPF intake between the 2 exams and tested for statistical significance using a paired *t*-test. This analysis was conducted to evaluate the extent of within-person tracking of UPF intake over the follow-up period and to inform the validity of using baseline dietary measures in long-term risk modeling. Cox proportional hazards models were used to evaluate the association between UPF consumption and ASCVD events. UPF consumption was analyzed as a continuous variable (servings/day), categorized into quintiles, and categorized into top 80% vs bottom 20%. Model 1 adjusted for sociodemographic factors (age, sex, race/ethnicity, education, and income). Model 2 additionally adjusted for key lifestyle-related confounders, including tobacco use, physical activity, and total energy intake in kilocalories per day; this was designated as the primary model for interpretation. Model 3 further adjusted for overall diet quality using the AHEI, and model 4 included additional cardiometabolic risk factors and potential mediators (diabetes history, high-density lipoprotein cholesterol, low-density lipoprotein cholesterol, lipid lowering medication use, systolic blood pressure, diabetes medication use, and waist-to-height ratio). Exposure and covariate data were assessed at baseline only, and outcomes were ascertained prospectively during follow-up using standardized MESA protocols. Models that included interactions for race/ethnicity, sex, and income were assessed and subgroup analyses stratifying the cohort were conducted for significant interactions to evaluate potential effect modification. Sensitivity analyses were conducted to address potential reverse causation by excluding participants with ASCVD events occurring within the first year of follow-up, and to assess the robustness of our findings to the exposure definition by testing a second variation of the UPF variable that examined UPF as a proportion of total servings/day (UPF servings/total servings), as well as using alternative versions of the UPF variable that excluded ambiguous or low-contribution items. In addition, restricted cubic splines were used to explore potential nonlinear relationships between UPF consumption and cardiovascular outcomes. In all analyses, *P* values <0.05 were considered statistically significant. STATA 18.5 was used for all analyses.

## Results

Baseline characteristics for MESA participants are displayed in Table 1. There were 3,438 females and 3,093 males. The sample included 39% White, 12% Chinese, 27% Black, and 22% Hispanic participants. In terms of SES, 65% of individuals had attained at least some college education or higher, whereas 32% reported a yearly total gross family income of less than $25,000, 46% had an income between $25,000 and $75,000, and 22% had an income exceeding $75,000. The average consumption of UPFs was 4.38 servings per day, ranging from 1.14 servings per day in the 1st quintile to 9.3 in the 5th quintile. Mean weight, waist circumference, and waist-to-height ratio increased across quintiles of UPF consumption (*P* trend <0.001). On average, UPFs accounted for 28% of the total daily food servings. UPFs constituted 41% of total daily servings among those in the 5th quintile of UPF consumption. Among Black Americans, UPFs constituted 32% of the total daily servings, compared to 30% among White, 24% among Hispanic, and 19% among Chinese Americans (*P* < 0.001).

**Table 1:** Baseline Characteristics of MESA Exam 1 Participants (N = 6,531)

	Ultraprocessed Food Intake Quintiles					
	1st Quintile (n = 1,312)	2nd Quintile (n = 1,305)	3rd Quintile (n = 1,302)	4th Quintile (n = 1,308)	5th Quintile (n = 1,304)	*P* Value
Ultraprocessed food intake (servings/day)	1.14 (0.46)	2.45 (0.35)	3.68 (0.39)	5.35 (0.59)	9.30 (3.11)	<0.001
UPF as a proportion of total servings/day (UPF servings/total servings)	0.17 (0.12)	0.23 (0.10)	0.27 (0.09)	0.32 (0.10)	0.41 (0.11)	<0.001
Age at exam 1	62.81 (10.09)	63.86 (10.19)	62.58 (10.20)	61.97 (10.12)	59.94 (10.15)	<0.001
Sex						<0.001
Female	788 (22.92)	732 (21.29)	698 (20.30)	620 (18.03)	600 (17.45)	
Male	524 (16.94)	573 (18.53)	604 (19.53)	688 (22.24)	704 (22.76)	
Race/ethnicity						<0.001
White	233 (9.15)	410 (16.10)	583 (22.90)	674 (26.47)	646 (25.37)	
Chinese American	442 (54.98)	221 (27.49)	78 (9.70)	35 (4.35)	28 (3.48)	
Black American	260 (14.86)	328 (18.74)	359 (20.51)	381 (21.77)	422 (24.11)	
Hispanic	377 (26.35)	346 (24.18)	282 (19.71)	218 (15.23)	208 (14.54)	
Weight (kg)	71.61 (16.33)	74.54 (15.91)	79.07 (16.29)	81.28 (16.27)	85.47 (17.65)	<0.001
Height (cm)	162.86 (9.35)	164.39 (9.72)	166.92 (9.78)	168.29 (9.76)	169.37 (10.01)	<0.001
Waist (cm)	94.13 (14.0)	96.12 (13.32)	98.09 (13.86)	99.13 (14.03)	102.50 (14.92)	<0.001
Waist to height ratio	0.58 (0.09)	0.59 (0.08)	0.59 (0.09)	0.59 (0.09)	0.61 (0.09)	<0.001
Education						<0.001
High school or less	591 (25.64)	493 (21.39)	454 (19.7)	375 (16.27)	392 (17.01)	
Some college or more	721 (17.06)	812 (19.21)	848 (20.07)	933 (22.08)	912 (21.58)	
HDL cholesterol (mg/dL)	51.74 (15.06)	51.75 (15.06)	51.40 (15.44)	50.93 (14.59)	49.17 (13.99)	<0.001
LDL cholesterol (mg/dL)	117.67 (32.47)	117.01 (31.86)	115.97 (30.59)	117.13 (30.95)	117.94 (30.82)	0.55
Any lipid lowering medication						0.60
No	1,093 (20.06)	1,083 (19.87)	1,078 (19.78)	1,093 (20.06)	1,103 (20.24)	
Yes	217 (20.34)	220 (20.62)	223 (20.90)	213 (19.96)	194 (18.18)	
Systolic blood pressure (mm Hg)	126.21 (22.83)	128.19 (21.82)	126.31 (21.14)	126.10 (20.87)	125.65 (20.16)	0.027
Any diabetes medication						0.013
No	1,157 (19.61)	1,173 (19.88)	1,185 (20.08)	1,184 (20.07)	1,201 (20.36)	
Yes	155 (24.56)	132 (20.92)	117 (18.54)	124 (19.65)	103 (16.32)	
Diabetes						0.035
No	1,095 (19.55)	1,110 (19.81)	1,118 (19.96)	1,131 (20.19)	1,148 (20.49)	
Yes	182 (22.98)	169 (21.34)	150 (18.94)	159 (20.08)	132 (16.67)	
Tobacco use (pack-years)	7.26 (15.87)	9.51 (17.84)	10.62 (19.03)	12.54 (21.72)	15.77 (26.86)	<0.001
Total moderate and vigorous physical activity (met-min/week)	4,842 (5,046)	5,175 (5,576)	5,651 (5,919)	6,212 (6,099)	6,661 (6,405)	<0.001

Continuous variables are mean (SD) or median (IQR) based on distribution, and categorical variables are n (%).

HDL = high-density lipoprotein; LDL = low-density lipoprotein; MESA = MultiEthnic Study of Atherosclerosis; UPF = ultraprocessed food.

UPF intake and its association with incident CVD events are presented in Table 2. During the follow-up, a total of 710 incident CVD events were ascertained among 6,531 participants, corresponding to 83,870 person-years of follow-up. In the sociodemographic-adjusted model (model 1), higher UPF consumption was associated with an increased risk of incident CVD (HR: 1.038; 95% CI: 1.011-1.064). This association persisted after further adjustment for total energy intake, physical activity, and tobacco use (model 2; HR: 1.051; 95% CI: 1.011-1.093). Additional adjustment for overall diet quality using the AHEI (model 3) and cardiometabolic risk factors (model 4) yielded similar results (HR: 1.047; 95% CI: 1.007-1.086 and HR: 1.052; 95% CI: 1.010-1.094, respectively). In sensitivity analyses excluding participants who experienced ASCVD events within the first year of follow-up, the associations between UPF intake and CVD risk remained materially unchanged (HR: 1.054; CI: 1.014-1.095). The Central Illustration depicts the dose-response association between UPF consumption (servings/day) and CVD risk. We observed a graded, approximately linear increase in CVD risk with each serving of UPF intake. At lower intakes, the hazard ratio was close to 1.0, but risk rose steadily with increasing UPF consumption, reaching nearly a 4-fold higher risk at 30 servings per day.

**Table 2:** Ultraprocessed Food Intake (Serving/Day) as a Predictor of CVD Events

	HR	95% CI	*P* Value
Model 1 (servings/day)	1.038	1.011-1.064	0.005
Model 1 (quintiles)			
2nd quintile	1.338	1.058-1.693	0.015
3rd quintile	1.398	1.103-1.773	0.006
4th quintile	1.383	1.084-1.764	0.009
5th quintile	1.481	1.157-1.894	0.002
Model 2 (servings/day)	1.051	1.011-1.093	0.011
Model 2 (quintiles)			
2nd quintile	1.274	1.003-1.617	0.047
3rd quintile	1.337	1.039-1.721	0.024
4th quintile	1.344	1.001-1.718	0.050
5th quintile	1.458	1.058-2.011	0.021
Model 3 (servings/day)	1.047	1.007-1.086	0.020
Model 3 (quintiles)			
2nd quintile	1.367	1.074-1.741	0.011
3rd quintile	1.396	1.081-1.804	0.011
4th quintile	1.419	1.074-1.874	0.014
5th quintile	1.517	1.088-2.116	0.014
Model 4 (servings/day)	1.052	1.010-1.094	0.014
Model 4 (quintiles)			
2nd quintile	1.374	1.077-1.753	0.010
3rd quintile	1.447	1.117-1.872	0.005
4th quintile	1.494	1.127-1.979	0.005
5th quintile	1.668	1.196-2.325	0.003
Model 4 (reference bottom 20%)			
Top 80%	1.410	1.133-1.754	0.002
Interaction model (UPF∗race)			
Chinese	1.086	0.927-1.273	0.304
Black	1.077	1.017-1.140	0.010
Hispanic	1.047	0.981-1.118	0.169
Model 4 stratified by Race			
Non-Black UPF (servings/day)	1.032	1.001-1.065	0.049
Black UPF (servings/day)	1.061	1.016-1.108	0.007

Cox models performed with ultraprocessed food as a continuous variable measured in servings per day and as a categorical variable in quintiles with the first quintile used as the reference group. Model 1 was adjusted for age, gender, race, education and income. Model 2 included model 1 plus tobacco use, physical activity, and total energy intake in kilocalories per day. Model 3 included model 2 plus diet quality assessed using the Alternative Healthy Eating Index (AHEI). Model 4 included model 2 plus diabetes history, high-density lipoprotein cholesterol, low-density lipoprotein cholesterol, lipid lowering medication use, systolic blood pressure, diabetes medication use, and waist-to-height ratio.

CVD = cardiovascular disease; other abbreviation as in Table 1.

**Central Illustration:**
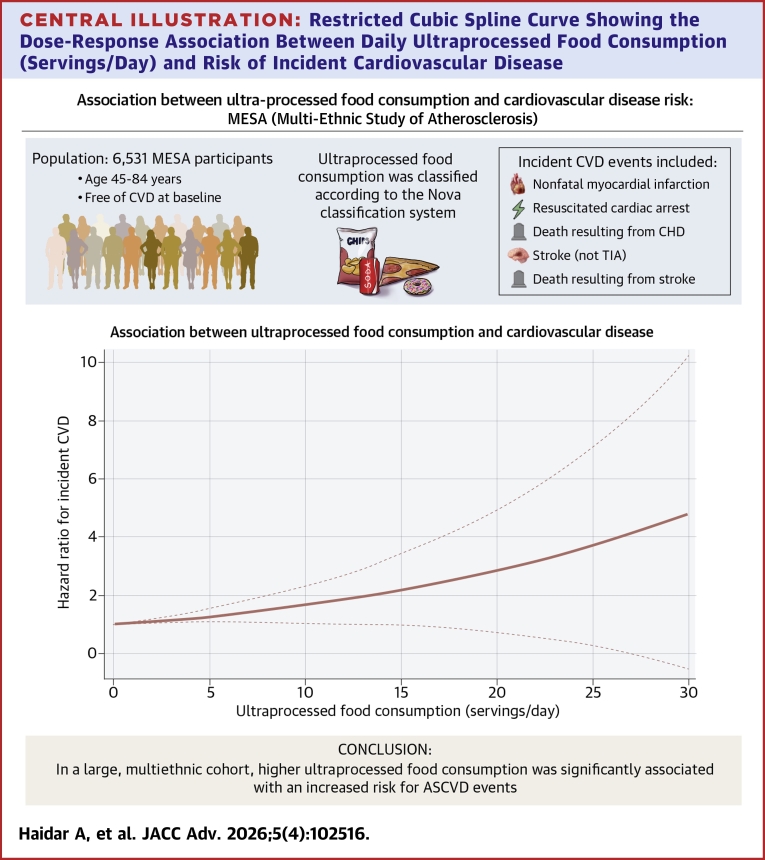
**Restricted Cubic Spline Curve Showing the Dose-Response Association Between Daily Ultraprocessed Food Consumption (Servings/Day) and Risk of Incident Cardiovascular Disease** The solid line represents the HR, and the dashed lines indicate 95% CIs. Higher UPF intake was associated with a progressively increased risk of CVD in MESA. ASCVD = atherosclerotic cardiovascular disease; CHD = coronary heart disease; CVD = cardiovascular disease; TIA = transient ischemic attack.

**Figure 1:**
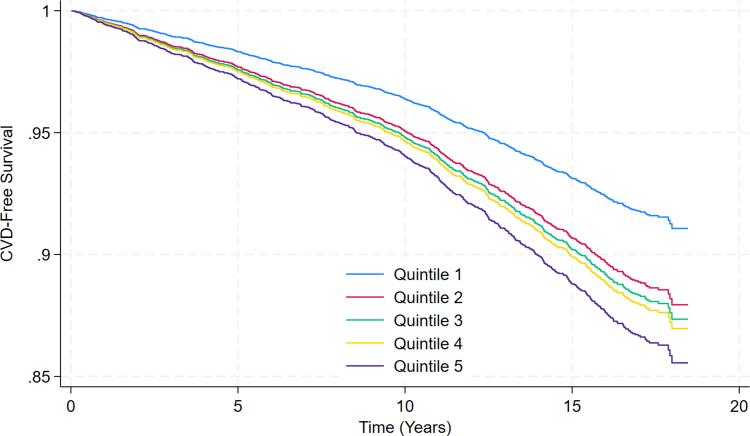
**Survival Curves for Incident Cardiovascular Disease Events by Quintiles of Ultraprocessed Food Intake (Servings/Day)** Participants were categorized into quintiles of daily UPF consumption. CVD-free survival progressively declined across higher quintiles of UPF intake, with the lowest survival observed in quintile 5 (highest UPF intake) and the highest survival in quintile 1 (lowest UPF intake) over a follow-up period of up to 20 years. CVD = cardiovascular disease.

When compared to those in the bottom 20% of UPF consumption, those in the top 80% had a 41.0% (HR: 1.410; CI: 1.133-1.754) increased risk of incident CVD events, whereas those in the highest 20% had a 66.8% (HR: 1.668; CI: 1.196-2.325) increase in risk (Figure 1). There was a significant multiplicative interaction between UPF intake and Black race (*P* = 0.010). When the fully adjusted model was stratified by race, Black Americans had a 6.1% increased risk of CVD events for every serving of UPF consumed, compared to a 3.2% increased risk among non-Black Americans. No significant interactions were observed between UPF intake, and sex or income.

When UPF intake was expressed as a proportion of total daily food servings, results were consistent with the primary analyses (Table 3). Each 10% increase in the proportion of UPF intake was associated with a higher risk of incident CVD events in all models: HR 1.105 (95% CI: 1.045-1.167) in model 1, 1.098 (95% CI: 1.038-1.163) in model 2, 1.093 (95% CI: 1.031-1.159) in model 3, and 1.101 (95% CI: 1.039-1.166) in model 4. Participants in the highest quintile proportion of UPF servings/total servings had a 50% greater risk of incident CVD compared with those in the lowest quintile (HR: 1.501; 95% CI: 1.170-1.925). A significant interaction was observed between UPF intake and race (*P* = 0.044). In stratified analyses, for every 10% increase in the proportion of UPFs in total daily food intake, Black Americans experienced a 12.3% increased risk of incident CVD events, compared with 7.9% increased risk among non-Black Americans. Exploratory analyses were conducted examining UPFs subdivided into categories and CVD risk (Supplemental Table 1). When UPF intake was analyzed by major food category, the direction of associations with CVD risk was generally positive but varied by food type. In fully adjusted models, higher consumption of sugary foods was significantly associated with greater CVD risk (HR: 1.12; CI: 1.03-1.22), whereas meats showed a borderline association (HR: 1.15; CI: 0.97-1.36). Bread and cereals were modestly associated with CVD risk in less-adjusted models, but this association was attenuated after full adjustment. No significant associations were observed for sugar-sweetened beverages, savory foods, or mixed dishes.

**Table 3:** Ultraprocessed Foods as a Proportion of Total Daily Food Servings as a Predictor of CVD Events

	HR	95% CI	*P* Value
Model 1 (10% increase UPF servings/total servings)	1.105	1.045-1.167	*P* < 0.001
Model 1 (quintiles)			
2nd quintile	1.139	0.898-1.444	0.283
3rd quintile	1.243	0.983-1.572	0.070
4th quintile	1.281	1.009-1.628	0.042
5th quintile	1.511	1.189-1.920	0.001
Model 2 (10% increase UPF servings/total servings)	1.098	1.038-1.163	0.001
Model 2 (quintiles)			
2nd quintile	1.130	0.889-1.436	0.317
3rd quintile	1.233	0.971-1.564	0.085
4th quintile	1.262	0.988-1.611	0.062
5th quintile	1.488	1.165-1.901	0.001
Model 3 (10% increase UPF servings/total servings)	1.093	1.031-1.159	0.003
Model 3 (quintiles)			
2nd quintile	1.125	0.884-1.429	0.337
3rd quintile	1.217	0.956-1.550	0.110
4th quintile	1.248	0.974-1.597	0.079
5th quintile	1.462	1.137-1.878	0.003
Model 4 (10% increase UPF servings/total servings)	1.101	1.039-1.166	0.001
Model 4 (quintiles)			
2nd quintile	1.109	0.869-1.414	0.405
3rd quintile	1.211	0.952-1.541	0.119
4th quintile	1.250	0.975-1.601	0.078
5th quintile	1.501	1.170-1.925	0.001
Interaction model (UPF∗race)			
Chinese	1.044	0.813-1.342	0.734
Black	1.159	1.004-1.337	0.044
Hispanic	1.125	0.969-1.305	0.121
Model 4 stratified by race			
Non-Black UPF (10% increase UPF servings/total servings)	1.079	1.008-1.155	0.028
Black UPF (10% increase UPF servings/total servings)	1.123	1.004-1.256	0.043

Cox models performed with ultraprocessed food as a proportion of total daily food servings in 10% increments and as a categorical variable in quintiles with the first quintile used as the reference group. Model 1 was adjusted for age, gender, race, education and income. Model 2 included model 1 plus tobacco use, physical activity, and total energy intake in kilocalories per day. Model 3 included model 2 plus diet quality assessed using the Alternative Healthy Eating Index (AHEI). Model 4 included model 2 plus diabetes history, high-density lipoprotein cholesterol, low-density lipoprotein cholesterol, lipid lowering medication use, systolic blood pressure, diabetes medication use, and waist-to-height ratio.

Abbreviations as in Table 1 and 2.

## Discussion

Prior studies have called on future research to address UPF consumption in diverse populations.^9^^,^^10^^,^^28^ This study addresses a gap in the literature as the first study to examine UPF consumption in a large, diverse, multiethnic population. The results from this prospective cohort analysis of MESA indicate that at baseline, UPF consumption was a significant predictor of incident ASCVD events. Participants with greater UPF consumption had higher risk of ASCVD events. Furthermore, the present study identified a significant multiplicative interaction between UPF consumption and race, indicating that race modifies the relationship between UPF intake and incident CVD.

The findings in this study are consistent with previous research investigating the association between UPF consumption and cardiovascular risk.^9^, ^10^, ^11^, ^12^, ^13^, ^14^, ^15^, ^16^, ^17^, ^18^, ^19^, ^20^, ^21^, ^22^, ^23^ Notably, a recent 2024 analysis of 3 large U.S. prospective cohorts, and systematic review and meta-analysis conducted by Mendoza et al.,^9^ found that total UPF consumption was adversely associated with CVD and CHD risk in U.S. adults, with findings corroborated by prospective studies from multiple countries. Our study corroborates findings from other U.S. cohorts, including the Framingham Offspring Study,^10^ Atherosclerosis Risk in Communities Study,^11^ and PLCO (Prostate, Lung, Colorectal, and Ovarian) Cancer Screening Trial^12^ which demonstrated similar results. Multiple international studies conducted in various, mostly European countries have also shown a strong positive association between UPF consumption and CVD risk.^13^, ^14^, ^15^, ^16^, ^17^, ^18^, ^19^, ^20^, ^21^, ^22^, ^23^ Although there is robust evidence to support the link between UPF consumption and CVD risk, prior studies have lacked racial and ethnic diversity, and the present study contributes to the evidence by demonstrating that these associations persist in a racially and ethnically diverse population.

An important finding in the present study was the interaction between race and UPF consumption. In our model, the interaction term for Black Americans was significant, indicating that the association of UPF consumption on incident CVD is stronger for Black Americans when compared to White Americans. In addition, when the model was stratified by race, the ASCVD risk was higher for Black Americans when compared to non-Black Americans. These results suggest that UPF consumption has a stronger effect on CVD risk in Black Americans than in other races or ethnicities. When compared to White, Chinese, or Hispanic Americans, Black Americans had the highest percentage of UPF in their diet and consumed more servings of UPF per day and less servings of non-UPF per day and had the highest percent of individuals in the fifth quintile of UPF consumption. Systemic factors rooted in historical racism, rather than inherent biological differences, may explain why UPFs have disproportionately negative health consequences for Black Americans. The higher burden of disease is largely driven by socioenvironmental factors and chronic stress, which may be exacerbated by UPF consumption.^25^^,^^46^^,^^47^ One contributing factor is that U.S. food companies disproportionately target Black and Hispanic consumers with marketing for primarily unhealthy, high-calorie, low-nutrient products, including candy, sugary drinks, snacks, chips, sugary cereals, and fast food.^30^ This contributes to the already pre-existing health disparities that exist among minorities and underscores the need for further research into potential disparities in dietary risk factors for CVD.

In subgroup analyses, sugary foods were the UPF category most consistently associated with a higher CVD risk, whereas other categories, including mixed dishes and processed meats, showed weaker associations. Prior research that examined UPF subgroups have reported mixed and inconsistent findings, highlighting the broader challenge of subgroup analyses in this area.^10^^,^^21^ Currently, there are no standardized classifications for UPF subgroups, and prior studies, including the present study, were not designed to fully account for this limitation, as most relied on FFQ data that were retrospectively categorized to create UPF variables. This lack of harmonization contributes to variability across studies and underscores a major limitation in the current literature. Future research should aim to refine UPF subgroup definitions by integrating information on food type, nutrient composition, and degree of processing to better characterize their distinct health impacts.

Several biological mechanisms and pathways have been observed to influence the relationship between UPF consumption and CVD.^5^^,^^10^ In the present study, those with higher UPF consumption had a greater intake of non-UPFs and higher waist-to-height ratios. UPFs might influence cardiometabolic health by increasing energy intake, altering the gut microbiome, interfering with gut-brain satiety signaling, and hormonal effects which then may lead to insulin resistance, dyslipidemia, obesity, hypertension, endothelial dysfunction, and oxidative stress.^5^ Higher circulating inflammatory protein biomarkers have also been found in those with higher UPF consumption, suggesting that inflammation pathways may explain the association between UPF consumption and CVD.^16^ In addition, UPF consumption has also been shown to be correlated with biomarkers representing renal function, liver function, inflammation, lipid metabolism, and glucose metabolism which in 11 study mediated the association between UFP consumption and CVD mortality.^18^ Future research is needed to further elucidate the harmful effects of ultraprocessing and the biological mechanisms through which UPFs affect CVD risk.

Our findings have important clinical and public health implications. Clinically, they highlight the need for health care providers to recognize UPF consumption as a potentially modifiable dietary risk factor for CVD, particularly among populations already experiencing a disproportionate burden of CVD.^48^^,^^49^ From a public health perspective, reducing UPF consumption will likely require strategies beyond individual behavior change, including policy interventions to improve the availability and affordability of healthier foods, regulation of targeted marketing of unhealthy products, and community-level initiatives to promote culturally tailored dietary patterns that emphasize minimally processed foods.^49^, ^50^, ^51^ Given the higher UPF intake and stronger associations with CVD risk observed in Black participants, interventions aimed at reducing structural barriers to healthy eating in historically marginalized communities may be especially impactful.^49^^,^^50^ Together, these findings underscore the importance of integrating dietary education, community resources, and policy-level action to mitigate the adverse health effects of UPFs and address diet-related health disparities.

There are many strengths of the present study as it is the first to examine UPF and CVD risk in a large, well-characterized multiethnic sample without clinically apparent CVD at baseline which lends to the generalizability of findings to a healthy population. Furthermore, the prospective study design and long-term follow-up establish temporality between UPF consumption and incident CVD risk. The MESA FFQ has been previously evaluated for reliability and validity in ethnically diverse populations. In addition, UPFs were classified according to a standardized and objective Nova classification system. Although dietary behaviors may change over time among participants with available dietary data at both exam 1 (2000-2002) and exam 5 (2010-2012), there was no statistically significant change in mean UPF intake over this 10-year interval. This finding demonstrates relative tracking of UPF consumption within individuals and supports the validity of baseline dietary assessment for estimating the long-term risk.

### Study Limitations

Limitations of the study include the inability to make causal inferences between UPF consumptions and CVD events. The FFQ data in this study were obtained from self-report, which could lead to under-reporting, failure to report, or exaggerated data. Furthermore, the FFQ data used were not explicitly developed to address questions related to food processing. Although the Nova classification was used to classify foods as ultraprocessed, misclassification of FFQ items cannot be excluded as inconsistencies with food assignments when applying the Nova criteria have been reported.^52^ Nevertheless, a systematic, multistep classification process was followed to ensure reproducibility and accuracy, and several versions of the UPF variable were developed, excluding ambiguous or low-contribution items, with findings remaining consistent. UPF intake was operationalized in servings per day, and the absence of energy- or weight-based measures represents a limitation given variation in serving size and caloric contribution across foods. Although significant associations between UPF intake and CVD risk were observed in both Black and non-Black participants, stratified analyses inherently reduce statistical precision. As a result, our ability to detect true differences in associations across racial/ethnic groups may have been limited, and these findings should be interpreted with caution.

## Conclusions

In a large, multiethnic cohort, higher UPF consumption was significantly associated with an increased risk for ASCVD events. Notably, this association was more pronounced among Black American participants, highlighting potential racial disparities in dietary risk factors for CVD. These findings contribute to the growing body of evidence linking UPF intake with adverse cardiometabolic health outcomes and underscore the need for targeted public health interventions and dietary guidelines aimed at reducing UPF consumption, particularly in vulnerable populations. Future research should further investigate the biological mechanisms underlying these associations and explore policy-driven strategies to mitigate the impact of UPFs on cardiovascular health.

Perspectives **COMPETENCY IN MEDICAL KNOWLEDGE:** High consumption of UPFs was associated with an increased risk of incident CVD in a large, diverse, multiethnic U.S. cohort. The observed associations were consistent across multiple analytic approaches and were stronger among Black participants, highlighting the importance of considering population subgroups when assessing dietary risk factors. These findings support recommendations to reduce UPF intake and reinforce the role of dietary quality as a modifiable determinant of cardiometabolic health.

**TRANSLATIONAL OUTLOOK:** Future research should investigate mechanisms linking UPFs to CVD, including the role of food additives, displacement of nutrient-dense foods, and contributions to metabolic dysfunction. Studies are needed to evaluate interventions aimed at reducing UPF consumption, particularly within populations disproportionately exposed due to structural and environmental factors. Clinical and policy efforts should focus on strategies that promote access to and affordability of healthier alternatives, while addressing barriers such as targeted marketing and retail food environments.

## Funding support and author disclosures

This research was supported by contracts 75N92020D00001, HHSN268201500003I, N01-HC-95159, 75N92020D00005, N01-HC- 95160, 75N92020D00002, N01-HC-95161, 75N92020D00003, N01-HC-95162, 75N92020D00006, N01-HC-95163, 75N92020D00004, N01-HC-95164, 75N92020D00007, N01-HC-95165, N01-HC-95166, N01-HC-95167, N01-HC-95168, and N01-HC-95169 from the 10.13039/100000050National Heart, Lung, and Blood Institute and by grant Nos. UL1-TR-000040, UL1-TR-001079, and UL1-TR-001420 from the 10.13039/100006108National Center for Advancing Translational Sciences. This publication was developed under the Science to Achieve Results research assistance agreements Nos. RD831697 (MESA Air) and RD-83830001 (MESA Air Next Stage) awarded by the U.S. Environmental Protection Agency. It has not been formally reviewed by the U.S. Environmental Protection Agency. The views expressed in this document are solely those of the authors, and the U.S. Environmental Protection Agency does not endorse any products or commercial services mentioned in this publication. The authors have reported that they have no relationships relevant to the contents of this paper to disclose.
