# Supplementary material for: Association Between Ultraprocessed Food Consumption and Cardiovascular Disease Risk: MESA (Multiethnic Study of Atherosclerosis)
Source: JACC Adv. 2026 Mar 17;5(4):102516. doi: 10.1016/j.jacadv.2025.102516 (PMC13131429; doi:10.1016/j.jacadv.2025.102516)
Supplement: Supplementary Tables 1 and 2 and Supplementary Figure 1 [file mmc1.docx]

| Supplementary Table 1: Ultra-processed food categories as a predictor of hard CVD Events at 10 Years (N = 6,531) | | | | | | | | | |
| --- | --- | --- | --- | --- | --- | --- | --- | --- | --- |
|  | Model 1 | | | Model 2 | | | Model 3 | | |
| UPF Categories | Hazard Ratio | 95% confidence interval | P-value | Hazard Ratio | 95% confidence interval | P-value | Hazard Ratio | 95% confidence interval | P-value |
| Bread and Cereals | 1.072 | 1.004, 1.145 | 0.037 | 1.069 | 1.002, 1.142 | 0.044 | 1.064 | 0.996, 1.135 | 0.065 |
| Sugar-Sweetened Beverages | 1.042 | 0.996, 1.091 | 0.077 | 1.039 | 0.992, 1.089 | 0.105 | 1.036 | 0.988, 1.086 | 0.142 |
| Sugary Foods | 1.087 | 0.996, 1.187 | 0.164 | 1.116 | 1.022, 1.219 | 0.014 | 1.120 | 1.025, 1.224 | 0.012 |
| Meats | 1.197 | 1.019, 1.405 | 0.028 | 1.180 | 1.001, 1.391 | 0.048 | 1.152 | 0.974, 1.362 | 0.099 |
| Savory Foods | 1.091 | 0.904, 1.318 | 0.361 | 1.107 | 0.884, 1.297 | 0.484 | 1.035 | 0.852, 1.258 | 0.725 |
| Mixed Dishes | 1.294 | 1.021, 1.641 | 0.033 | 1.255 | 0.984, 1.600 | 0.067 | 1.158 | 0.897, 1.495 | 0.259 |
| Cox models performed with ultra-processed food as a proportion of total daily food servings in 10% increments and as a categorical variable in quintiles with the first quintile used as the reference group. Model 1 was adjusted for age, gender, race, education and income. Model 2 included model 1 plus tobacco use, physical activity, diabetes history, and lipid lowering medication use. Model 3 included model 2 plus high-density lipoprotein cholesterol, low-density lipoprotein cholesterol, systolic blood pressure, and diabetes medication use. Bread and cereals consisted of hot cereal, cold cereal, white bread, dark bread, muffins, and biscuits. Sugar-sweetened beverages consisted of sweet milk, soy milk, soda, diet soda, instant breakfast, and hot cocoa. Sugary foods consisted of ice cream, frozen yogurt, tofu dessert, white donuts, chocolate donuts, pancakes, pies, pudding, and candy. Meats consisted of hamburgers, hamhocks, fried chicken, sausage and fried fish. Savory foods consisted of chips and crackers. Mixed dishes consisted of french fries, oriental noodles, dumplings, chow mein, refried beans, and pizza. | | | | | | | | | |

| Supplementary Table 2. Mapping FFQ Food Items Classified as Ultra-Processed Foods | | |
| --- | --- | --- |
| FFQ Item | Example Description | Rationale for Classification |
| Sausage | Store-bought or processed breakfast sausage | Industrially formulated meat product with preservatives, flavorings, and curing agents |
| Pancakes | Packaged or instant mix | Refined-grain mix with added sugars, emulsifiers, and flavorings |
| Hot cereal | Instant flavored oatmeal | Pre-flavored and sweetened grain product with additives |
| Cold cereal | Sweetened ready-to-eat cereal | Extruded grain product with added sugars, flavorings, and stabilizers |
| White bread | Packaged refined white bread | Refined flour, emulsifiers, preservatives, and added sugars |
| Dark bread | Packaged wheat or multigrain bread | Industrial formulation with added sugars, oils, and preservatives |
| Muffins | Packaged or commercial muffins | Refined-grain baked good with added sugars, fats, and stabilizers |
| Biscuits | Packaged or refrigerated biscuit dough | Refined-grain dough with hydrogenated oils and preservatives |
| Margarine on rolls | Industrial margarine spread | Fat-based spread with emulsifiers, colorants, and flavorings |
| Chips | Packaged potato or corn chips | Fried or baked snack with added flavorings, oils, and preservatives |
| Crackers | Packaged savory crackers | Refined-grain snack with hydrogenated oils and flavorings |
| French fries | Frozen or fast-food fries | Industrially prepared with oils, salt, and preservatives |
| Oriental noodles | Instant noodles with seasoning packet | Dried noodle with flavor packet containing additives and preservatives |
| Dumplings | Frozen pre-packaged dumplings | Multi-ingredient industrial product with additives and flavorings |
| Chow mein | Packaged or frozen chow mein | Ready-to-heat dish with sauces and additives |
| Refried beans | Canned refried beans | Industrial formulation with added oils and stabilizers |
| Pizza | Frozen or fast-food pizza | Multi-ingredient product with preservatives and flavorings |
| Hamburger | Fast-food hamburger | Processed meat and refined-grain bun with additives |
| Hamhocks | Smoked or cured hamhocks | Cured and preserved meat with nitrates and flavoring agents |
| Fried chicken | Fast-food fried chicken | Industrially prepared with batter, oils, and preservatives |
| Fried fish | Frozen or fast-food breaded fish | Breaded, fried product with preservatives and flavorings |
| Ice cream | Commercial packaged ice cream | Dairy-based dessert with added sugars, emulsifiers, and stabilizers |
| Frozen yogurt | Commercial frozen yogurt | Sweetened dairy dessert with stabilizers and flavorings |
| Tofu dessert | Packaged sweetened tofu pudding | Soy-based sweetened dessert with stabilizers |
| White donuts | Packaged donuts | Refined-grain baked good with sugars, fats, and additives |
| Chocolate donuts | Packaged donuts | Same as above, with cocoa flavoring and added sugars |
| Pies | Packaged fruit pie | Refined flour crust, added sugars, oils, and stabilizers |
| Pudding | Instant pudding mix | Sweetened, flavored product with emulsifiers and stabilizers |
| Candy | Packaged confectionery | Industrially manufactured confection with additives |
| Sweet milk | Flavored milk | Milk with added sugar and stabilizers |
| Soy milk | Flavored or sweetened soy beverage | Soy beverage with flavoring and stabilizers |
| Soda | Sugar-sweetened soft drink | Carbonated beverage with colorants and preservatives |
| Diet soda | Artificially sweetened soft drink | Carbonated beverage with non-nutritive sweeteners and flavorings |
| Instant breakfast | Powdered meal replacement drink | Powdered beverage mix with flavorings and stabilizers |
| Hot cocoa | Powdered cocoa drink mix | Powdered mix with added sugars and flavorings |
| Liquor | Distilled alcoholic beverage | Industrially distilled product sometimes with flavoring or coloring agents |

Supplementary Figure 1: Directed Acyclic Graph (DAG) showing hypothesized relationships between ultra-processed food intake and cardiovascular disease.


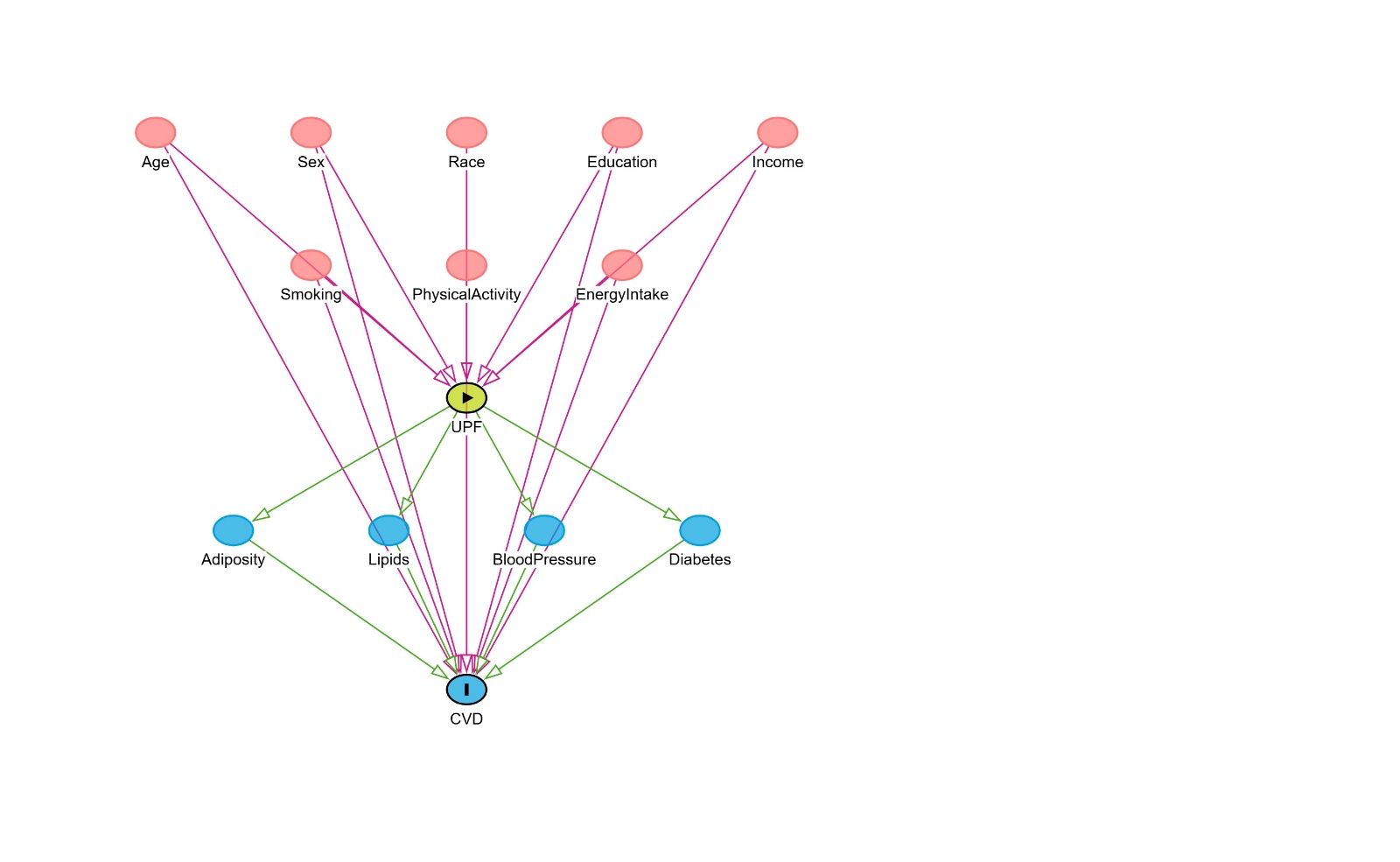


Directed acyclic graph (DAG) illustrating hypothesized relationships between ultra-processed food (UPF) intake and cardiovascular disease (CVD). UPF intake may influence CVD risk both directly and indirectly through cardiometabolic mediators, including adiposity, lipids, blood pressure, and diabetes. Confounding factors (age, sex, race/ethnicity, education, income, smoking, physical activity, total energy intake) were adjusted for in Model 2. Model 3 additionally included potential mediators to estimate controlled direct effects.
